# Development of molecular assays for the rapid and cost-effective determination of fluoroquinolone, macrolide and lincosamide susceptibility of *Mycoplasma synoviae* isolates

**DOI:** 10.1371/journal.pone.0241647

**Published:** 2020-10-30

**Authors:** Katinka Bekő, Zsuzsa Kreizinger, Cécile Yvon, Orsolya Saller, Salvatore Catania, Anneke Feberwee, Miklós Gyuranecz

**Affiliations:** 1 Institute for Veterinary Medical Research, Centre for Agricultural Research, Budapest, Hungary; 2 Instituto Zooprofilattico Sperimentale delle Venezie, Verona, Italy; 3 GD Animal Health Service, Deventer, The Netherlands; 4 Department of Microbiology and Infectious Diseases, University of Veterinary Medicine, Budapest, Hungary; Nitte University, INDIA

## Abstract

*Mycoplasma synoviae* infection occurs worldwide, leading to considerable economic losses in the chicken and turkey industry due to infectious synovitis, respiratory diseases and eggshell apex abnormalities. Control programs against *M*. *synoviae* infection are based on eradication, vaccination and medication with antimicrobial agents. Prudent use of antibiotics can be improved greatly by the determination of antibiotic susceptibility prior to the treatment. However, the conventional broth or agar microdilution is very labor-intensive and time-consuming method. Thus, there is an increasing need for rapid antimicrobial susceptibility tests in order to guide antibiotic therapy more effectively. The aim of this study was to develop mismatch amplification mutation assays (MAMAs) to detect resistance-associated mutations in *M*. *synoviae*. *M*. *synoviae* strains with previously determined minimal inhibitory concentrations (MICs) and whole genomes (n = 92) were used for target selection and assay specification. For the evaluation of the developed assays, 20 clinical samples and an additional 20 *M*. *synoviae* isolates derived from these specimens were also included in this study. MIC values of these 20 isolates were determined by broth microdilution method. Five MAMAs were designed to identify elevated MICs of fluoroquinolones, while three MAMAs were developed to detect decreased susceptibility to macrolides and lincomycin. The sensitivity of the MAMA tests varied between 10^2^−10^4^ template copy number/reaction depending on the assay. Clinical samples showed identical genotype calls with the *M*. *synoviae* isolates derived from the corresponding specimens in each case. Supporting the results of conventional *in vitro* sensitivity tests, our approach provides a feasible tool for diagnostics. Rapidity, robustness and cost-effectiveness are powerful advantages of the developed assays. Supporting prudent antibiotic usage instead of empirical treatment, the use of this method can reduce significantly the economic impact of *M*. *synoviae* in the poultry industry and decrease bacterial resistance-related public health concerns.

## Introduction

*Mycoplasma synoviae* infection occurs worldwide, leading to infectious synovitis or respiratory diseases in chickens and turkeys, and it can be related to eggshell apex abnormalities in chickens as well. In the affected flocks reduced feed intake, weight gain, egg production and hatchability can be observed, leading to great economic losses. Control programs against *M*. *synoviae* infection are based on eradication, vaccination and medication with antimicrobial agents [1].

Prudent use of antibiotics in the management of *M*. *synoviae* infection can be improved greatly by the determination of antibiotic susceptibility prior to the treatment. Most common method of antibiotic susceptibility testing is the determination of minimal inhibitory concentration (MIC) values *in vitro* by broth or agar microdilution. However, interpretation of the results is difficult, because neither standard breakpoints of susceptible, intermediate and resistant categories to antimicrobial agents, nor internationally harmonized testing conditions have been defined yet concerning avian *Mycoplasma* species. Moreover, the results of *in vitro* antibiotic susceptibility tests can only predict the expected *in vivo* efficacy of the antibiotics [2], and the microdilution tests are very labor-intensive and time-consuming methods, as they require previous isolation and pure culture of the bacterium [3]. Thus, there is an increasing need for rapid antimicrobial susceptibility tests in order to support the choice of the most appropriate antibiotic therapy. Investigating the correlation between *in vitro* antibiotic susceptibility and resistance-associated mutations can provide an alternative option, as the majority of the resistance mechanisms are mediated by genetic alterations [4]. A rapid and cost-effective method is the detection of resistance-associated mutations by molecular biological assays [5, 6].

Numerous mutations related to antibiotic-resistance have been already described in avian mycoplasmas, including *M*. *synoviae* as well. Several studies suggest that the primary target of fluoroquinolones in *M*. *synoviae* is the A subunit of the DNA topoisomerase IV enzyme (ParC) [7–9]. Mutations at the nucleotide positions 253–265 (or 238–250 according to *Escherichia coli* numbering) of the quinolone resistance-determining region (QRDR) can result in the alterations of ParC at the amino acid positions 85–89 (or 80–84 according to *E*. *coli* numbering) [7–9]. Beside this hot spot region, potentially resistance-related mutations have been identified in the *gyrA* (G28A, A428G, A566G, T1360A, C1361A, G1651A), *gyrB* (C446T, C1247A, G1250A), and *parE* (C260T) genes of *M*. *synoviae* isolates with higher MIC values for fluoroquinolones as well [7–9].

Mutations in the central loop of the domain V (peptidyl transferase region) of 23S rRNA confer resistance to 50S inhibitors in many bacteria. In *M*. *gallisepticum*, mutations at nucleotide positions 2057–2059 (according to *E*. *coli* numbering) of the 23S rRNA coding genes (*rrlA/B*) can lead to a disruption of the rRNA structure, thus alterations in this area can prevent the attachment of the antimicrobial agents to their binding site [10]. Mutations A2054G and A2055G (or 2058 and 2059 according to *E*. *coli* numbering) have been previously associated with decreased susceptibility for macrolides and lincomycin in *M*. *synoviae* as well [9, 11]. It also has been described, that resistance to lincomycin and tilmicosin does not require the A2054G mutation in both *rrl* genes in *M*. *synoviae*, as the presence of this mutation only in the *rrlA* or *rrlB* gene is enough to increase the MIC values of these antibiotics [9, 11].

Non-synonymous mutations of the *rplV* gene A276C/T (or 270 according to *E*. *coli* numbering) described previously in *M*. *synoviae* strains by Lysnyansky et al. [11] were also found to decrease the susceptibility for macrolides, especially in case of tilmicosin [9]. These single nucleotide polymorphisms (SNPs) result in a glutamine-histidine amino acid change at the position 92 (or 90 according to *E*. *coli* numbering) of the 50S ribosomal protein L22.

In the recent years, several studies found high MIC values of macrolides and lincosamides against *M*. *synoviae* isolates [11–13]. Susceptibility of *M*. *synoviae* isolates to fluoroquinolones also decreased over the last few decades, which is particularly troublesome as the use of fluoroquinolones is critical in the therapy of humans as well [7, 8, 12, 13].

The aim of this study was to develop rapid and cost-effective molecular biological assays for the detection of the most important fluoroquinolone, macrolide and lincosamide resistance-associated mutations in *M*. *synoviae*.

## Materials and methods

### Sample processing

In the present study, DNA samples of 20 clinical specimens and 112 *M*. *synoviae* strains, including the *M*. *synoviae* type strain NCTC 10124, the MS-H (Vaxsafe® MS, Bioproperties Pty Ltd., Ringwood, Australia) and MS1 (Nobilis® MS Live, MSD Animal Health Hungary, Budapest, Hungary) vaccine strains, 89 field isolates from previous studies [9, 12] and 20 *M*. *synoviae* isolates derived from the 20 clinical specimens were investigated. Samples were collected between 1982 and 2019, originated from 23 different countries, and were isolated from tracheal swabs or lungs of chickens and turkeys. According to the written declaration (reference number: IVMR/2019/0027) of the Ethics Committee of the Institute for Veterinary Medical Research, Centre for Agricultural Research ethical approval was not required for the study as the samples were taken during routine diagnostic examinations with the written consent of the owner. The final study and the manuscript was submitted and approved in a written declaration by the Ethics Committee of the Institute for Veterinary Medical Research, Centre for Agricultural Research. Background information of the used *M*. *synoviae* strains and clinical samples are provided in S1 Table.

*M*. *synoviae* culture was performed as described previously [9]. In brief, the collected samples were placed into liquid Frey’s media [14] (Sigma-Aldrich Inc., St. Louis, USA) and transported to the laboratory for incubation at 37°C in an atmosphere of 5% CO_2_. Following color change (red to yellow shift) of the phenol red due to the metabolic activity of mycoplasmas, the culture was inoculated onto solid Frey’s media [14] (Sigma-Aldrich Inc.) and incubated at 37°C in an atmosphere of 5% CO_2_ until visible colonies appeared. Filter cloning was performed to gain pure cultures from the isolates. When it was possible, cultures were filter cloned only once to minimize *in vitro* mutations of the isolates.

DNA extraction from 200 μl pure *M*. *synoviae* logarithmic-phase broth culture was performed using the ReliaPrep™ gDNA Tissue Miniprep System (Promega Inc., Madison, USA) according to the manufacturer’s instructions for Gram-negative bacteria. In order to confirm *M*. *synoviae* positivity of the samples, DNAs were submitted to *M*. *synoviae*-specific polymerase chain reaction (PCR) [15]. The presence of other, contaminant mycoplasmas (i.e. *M*. *gallisepticum*) was investigated by a universal *Mycoplasma* PCR system targeting the 16S/23S rRNA intergenic spacer region of Mollicutes [16]. The PCR products were subjected to Sanger sequencing on an ABI Prism 3100 automated DNA sequencer (Applied Biosystems, Foster City, USA) and sequences were submitted to Nucleotide BLAST search (http://www.ncbi.nlm.nih.gov/BLAST).

The DNA samples of the clinical specimens (original broth (tracheal swabs or lung tissue samples washed into Frey’s medium [14] (Sigma-Aldrich Inc.)) and FTA cards) were extracted using the Qiamp DNA Mini kit (Qiagen Inc., Hilden, Germany) according to the manufacturer’s instructions. The *M*. *synoviae* positivity and the presence of other, contaminant mycoplasmas were investigated by the aforementioned PCRs [15, 16].

### Broth microdilution

Minimal inhibitory concentration (MIC) values of two fluoroquinolones (enrofloxacin and difloxacin), three macrolides (tylosin, tilmicosin and tylvalosin) and a lincosamide (lincomycin) against 92 *M*. *synoviae* strains were determined previously by broth microdilution method [9, 12] according to the recommendation of Hannan [3]. In this study, an additional 20 *M*. *synoviae* isolates derived from the 20 examined clinical samples were submitted to susceptibility testing.

The antibiotics originated from VETRANAL (Sigma-Aldrich Chemie GmbH., Taufkirchen, Germany) except for tylvalosin (Aivlosin), which was purchased from ECO Animal Health Ltd. (London, UK). The antibiotics were diluted and stored according to the recommendations of Hannan [3]. Stock solutions of 1 mg/ml for fluoroquinolones were prepared in 0.1 M NaOH, while stock solutions of 1 mg/ml for macrolides and lincomycin were prepared in sterile distilled water and stored at -70°C. Freshly prepared two-fold dilutions were used in each microtest after checking the thawed antibiotic solutions for any visible changes in their consistency. The examined concentration range of the antibiotics was 0.039–10 μg/ml for fluoroquinolones and 0.25–64 μg/ml for macrolides and lincomycin.

Accepted number of microorganisms for the MIC tests was 10^4^−10^5^ color changing unit (CCU/ml). The duplicates of clinical isolates and the duplicate of the *M*. *synoviae* type strain NCTC 10124 were tested on each plate. The reference strain was included in the tests to confirm the validity of the results. The microtiter plates were sealed with adhesive film and incubated at a temperature of 37°C. The MIC value against each isolate was defined as the lowest concentration of the antibiotic that completely inhibited the growth in the broth, i.e. no color change has been observed. The MICs were read daily and recorded as soon as the growth controls changed color.

Elevated MIC values indicating decreased susceptibility of *M*. *synoviae* isolates were determined above 1.25 μg/ml for fluoroquinolones, 0.5 μg/ml for tylvalosin, 1 μg/ml for tylosin, 8 μg/ml for tilmicosin, and 2 μg/ml for lincomycin, based on our previous study [9].

### Assay development

For the detection of SNPs related to decreased susceptibility of *M*. *synoviae* isolates for fluoroquinolones, macrolides and lincomycin, mismatch amplification mutation assays (MAMAs) were designed. MAMA is a PCR-based technique used for SNP discrimination in many bacteria [17]. In brief, the technique is based on SNP-specific primers, one being marked with an additional 14–20 base pair (bp) long GC-clamp. The GC-clamp increases the melting temperature (Tm) of the amplicon, and this temperature shift can be easily detected in the presence of intercalating fluorescent dye on a real-time PCR platform (melt-MAMA).

Target mutations were selected according to the results of our previous study [9] based on their correlation with elevated MIC values. Besides, the targeted SNPs had to be surrounded by conserved regions suitable for primer design.

Primers were designed by Geneious software version 10.2.3. (Biomatters Ltd., Auckland, New Zealand) [18] based on the whole genome sequences (Sequence Read Archive (SRA) accession numbers: PRJNA634246 and PRJNA634252; GenBank accession numbers: CP011096 and KP704286) of the 92 previously examined *M*. *synoviae* strains [9]. Each primer set consisted of a consensus reverse primer and two competing forward primers designed to specifically target the resistance-related SNP. At the allele-specific 3′ end of the competing primers, a single antepenultimate destabilizing mismatch was inserted to enhance the discriminative capacity of the assay. The primer specific for the resistance-related SNP at the 3’ end was marked with an additional 14 bp long GC-clamp at the 5’ end. The primers were constructed to limit amplicon lengths of ≤120 bp. The general suitability of the designed primer sets was calculated by using NetPrimer software (Premier Biosoft International, Palo Alto, USA) (http://www.premierbiosoft.com/netprimer). The specificity of the primers was analyzed *in silico* using Nucleotide BLAST search (http://www.ncbi.nlm.nih.gov/BLAST).

The melt-MAMA PCR mixture consisted of nuclease-free water, 2 μl 5X Colorless GoTaq Flexi Buffer (Promega Inc.), 1 μl MgCl_2_ (25 mM; Promega Inc.), 0.3 μl dNTP (10 mM; Fermentas, Waltham, USA), 0.5 μl EvaGreen (20X, Biotium Inc., Hayward, USA), 0.08 μl GoTaq G2 Flexi DNA polymerase (5 U/μl; Promega Inc.), 1 μl target DNA solution and primers according to Table 1 with a final volume of 10 μl. Thermocycling parameters were 95°C for 10 minutes, followed by 35 cycles of 95°C for 15 seconds and 60°C for 1 minute. PCR products were subjected to melt analysis using a dissociation protocol comprising 95°C for 15 seconds, followed by 0.3°C incremental temperature ramping from 60°C to 95°C. The real-time PCRs were performed using Applied Biosystems Step-One Plus real-time PCR system with StepOne software version 2.3 (Thermo Fisher Scientific Inc., Waltham, USA). EvaGreen fluorescence intensity was measured at 525 nm at each ramp interval and plotted against temperature. Nuclease-free water was used as negative control in all PCR assays.

**Table 1:** Sequences of the designed primers and their volumes used in each assay.

Assay	Primer name^a^	Primer sequence (5’-3’)	Primer volume^b^
**MAMA-MS- gyrA**	gyrA-H	ggggcggggcggggCTAGAATGGCTAAAATTTCAACTGG	0.15
	gyrA-L	CTAGAATGGCTAAAATTTCAACGGA	0.15
	gyrA-C	CGGTTGAATCATAGTTATCGACAAA	0.15
**MAMA-MS- gyrB**	gyrB-H	ggggcggggcggggTTACAAAAGAAAAAAGGATTAGGTAACTA	0.15
	gyrB-L	TTACAAAAGAAAAAAGGATTAGGTAAATC	0.6
	gyrB-C	TTTTTACTTGAACAATCAGAAAGCTT	0.15
**MAMA-MS- parC-1**	parC-1-H	ggggcggggcggggAAGTATCAYCCTCATGGAGACAT	0.15
	parC-1-L	AAGTATCAYCCTCATGGAGAAAC	0.6
	parC-C	AATATTCATTTTTCATCACTGCG	0.15
**MAMA-MS- parC-2**	parC-2-H	ggggcggggcggggAAGTATCAYCCTCATGGAGCTG	0.15
	parC-2-L	AAGTATCAYCCTCATGGAGGTA	0.15
	parC-C	AATATTCATTTTTCATCACTGCG	0.15
**MAMA-MS- parE**	parE-H	ggggcggggcggggCAGTTGAAAAAGTYAAAGGTGAGTT	0.15
	parE-L	CAGTTGAAAAAGTYAAAGGTGATTC	0.15
	parE-C	TCCTGAGGTTTTATATGAATCAGAAG	0.15
**MAMA-MS- rrl**	rrl-H	ggggcggggcggggGGTACCCGCATCAAGACCAG	0.6
	rrl-L	GGTACCCGCATCAAGACAAA	0.15
	rrl-C	CACATGTTAGGCCAAATTTCAATA	0.15
**MAMA-MS- rplV-1**	rplV-H-1	ggggcggggcggggTTTAAAATTTCGTAAGCTCTTACG	0.3
	rplV-L	TTTAAAATTTCGTAAGCTCTTGCT	0.15
	rplV-C	ATGTAAATGAAGGTCCTACCTTAAA	0.15
**MAMA-MS- rplV-2**	rplV-H-2	ggggcggggcggggTTTAAAATTTCGTAAGCTCTTTCA	0.3
	rplV-L	TTTAAAATTTCGTAAGCTCTTGCT	0.15
	rplV-C	ATGTAAATGAAGGTCCTACCTTAAA	0.15

^a^H refers to: specific for the genotype characterized by high MIC values for certain antibiotics; L refers to: specific for the genotype characterized by low MIC values for certain antibiotics; C refers to: consensus

^b^Primer (10 pmol/μl) volume in 10 μl reaction mixture (μl)

### Specification and evaluation of the assays

Specification of the designed MAMAs was performed on DNAs extracted from pure cultures of the 92 previously examined *M*. *synoviae* strains [9]. Genotype calls of these strains (genotype H: genotype characterized by high MIC values for certain antibiotics; genotype L: genotype characterized by low MIC values for certain antibiotics; genotype Het: heterozygous concerning nucleotide position 2054 of the *rrl* genes) were compared with their whole genome sequences (SRA accession numbers: PRJNA634246 and PRJNA63425; GenBank accession numbers: CP011096 and KP704286).

In order to test the sensitivity of the assays, tenfold dilutions of each genotype were used in the range of 10^6^−10^0^ template copy number/μl. Template copy number was calculated with the help of an online tool [19] (http://cels.uri.edu/gsc/cndna.html) based on the DNA concentration measured by Nanodrop 2000 Spectrophotometer (Thermo Fisher Scientific Inc.). The lowest template copy number yielding melting temperature specific to the genotype was considered as the detection limit of each assay.

The specificity of the assays was tested using the following avian *Mycoplasma* species: *M*. *anatis* (ATCC 25524), *M*. *anseris* (ATCC 49234), *M*. *anserisalpingitidis* (ATCC BAA-2147), *M*. *cloacale* (ATCC 35276), *M*. *columbinasale* (ATCC 33549), *M*. *columborale* (ATCC 29258), *M*. *gallinaceum* (ATCC 33550), *M*. *gallinarum* (ATCC 19708), *M*. *gallopavonis* (ATCC 33551), *M*. *iners* (ATCC 19705), *M*. *imitans* (ATCC 51306), *M*. *iowae* (ATCC 33552), *M*. *meleagridis* (NCTC 10153), and *M*. *gallisepticum* (ATCC 19610).

For further evaluation of the assays, the designed MAMA tests were challenged with the DNAs of clinical specimens (n = 20) and *M*. *synoviae* isolates derived from these clinical samples (n = 20), and their genotype calls were compared to each other.

The false result rate of the tests was calculated for each examined antibiotic group. The tests were considered to give a false result if the genotype assignment of the applied assays were not in line with the MIC data of the tested strain, i.e. all of the assays resulted in melting curve specific for genotype L in isolates with elevated MIC value for at least one of the tested agents, or at least one of the assays resulted in melting curve specific for genotype H (or Het in case of the MAMA-MS-rrl) in strains with low MIC value for all of the tested agents.

## Results

Two MAMAs were designed to detect the SNPs A253G and C254T of the *parC* gene, resulting in amino acid change at position 85 of the ParC, which is the most relevant alteration concerning fluoroquinolone susceptibility of *M*. *synoviae* according to the results of our previous study [9]. Three additional resistance-related mutations of the *gyrA* (A566G), *gyrB* (C1247A) and *parE* (C260T) genes were also targeted based on their frequent occurrence [9].

To identify *M*. *synoviae* isolates with decreased susceptibility for macrolides and lincomycin, a MAMA was designed to detect the SNP at nucleotide position 2054 of the *rrlA/B* genes. Besides, for the determination of macrolide susceptibility, the mutation A276C/T of the *rplV* gene was also targeted by two assays.

During specification, genotype calls by the designed MAMAs were congruent with the whole genome sequences for all DNAs isolated from pure cultures of *M*. *synoviae* strains, except for the isolate MYCAV102, which was undetectable in five out of eight assays (S1 Table). The presented MAMAs clearly differentiated the two genotypes characteristic for *M*. *synoviae* isolates with high (genotype H) and low (genotype L) MIC values for certain antibiotics, based on their distinguishable peaks of melting curves (Table 2).

**Table 2:** Results of MAMAs designed in this study.

Antibiotics	Assay	SNP	AA subst.	Gt	Sensitivity (template copy number/ reaction)	Size of amplicons (bp)	Tm range of amplicons (°C)	Average Tm of negative control (°C)	Tm of cross-reacting *Mycoplasma spp*. (°C)
**FLUORO- QUINOLONES**	MAMA-MS-gyrA	A566G	Glu189Gly	H	10^2^	98	78.31–78.61	-	-
				L	10^2^	84	73.99–74.59		
	MAMA-MS-gyrB	C1247A	Ser416Tyr	H	10^2^	82	77.86–78.32	-	-
				L	10^3^	68	72.36–73.4		
	MAMA-MS-parC-1	C254T	Thr85Ile	H	10^2^	89	79.66–81.29	71.09	-
				L	10^2^	75	75.48–77.12		
	MAMA-MS-parC-2	A253G	Thr85Ala	H	10^3^	89	82.33–82.47	-	-
				L	10^3^	75	75.33–77.41		
	MAMA-MS-parE	C260T	Ser87Phe	H	10^2^	118	81.58–82.18	-	-
				L	10^3^	104	78.16–78.9		
**MACROLIDES, LINCOMYCIN**	MAMA-MS- rrl	A2054G	n.a.	H	10^3^	88	82.48–82.78	-	*M*. *anatis*: 77.12 *M*. *anserisalpingitidis*: 77.12 *M*. *gallinarum*: 76.38
				Het	10^2^	74; 88	77.27–77.42; 82.42–82.57		
				L	10^3^	74	77.12–77.72		
**MACROLIDES**	MAMA-MS-rplV-1	A276C	Gln90His	H	10^3^	85	79.35–79.5	84.42	-
				L	10^4^	71	74.44–75.63		
	MAMA-MS-rplV-2	A276T	Gln90His	H	10^3^	85	79.06–79.21	-	-
				L	10^4^	71	74.59–75.63		

SNP: single nucleotide polymorphism; AA subst.: amino acid substitution; Gt: genotype; bp: base pair; Tm: melting temperature; H: genotype characterized by high MIC values; L: genotype characterized by low MIC values; Het: heterozygous genotype

**Fig 1:**
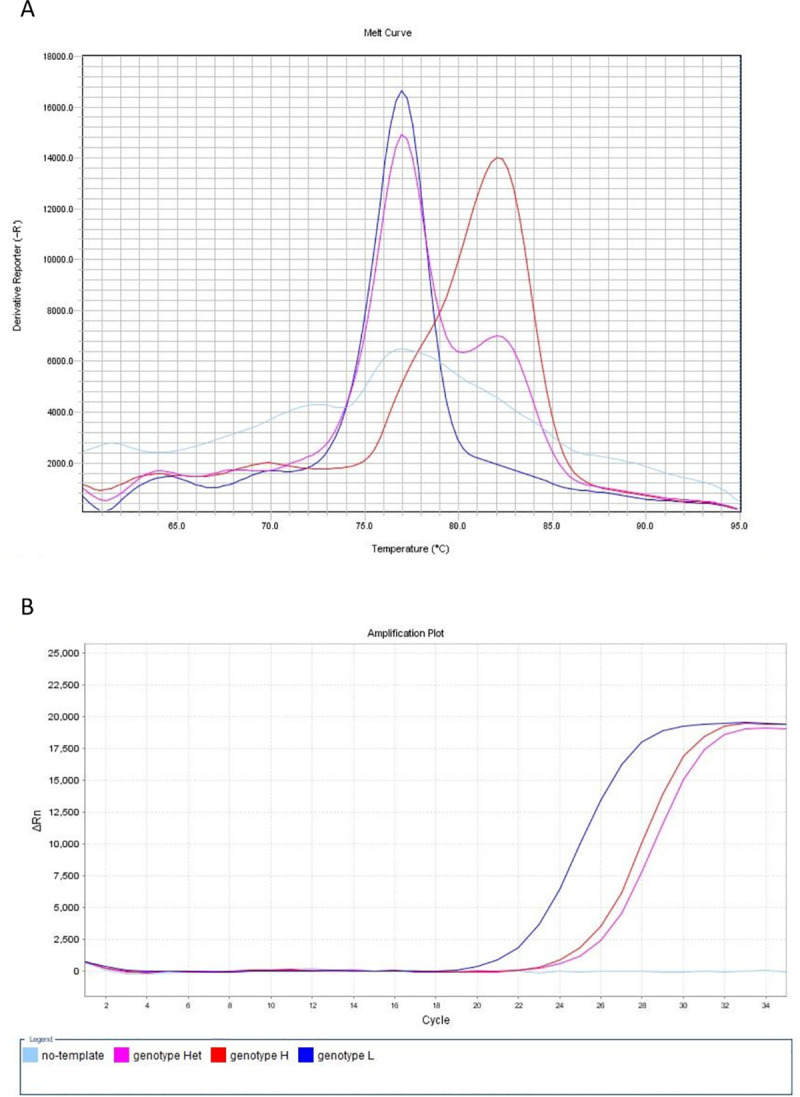
Melt curve (A) and amplification plot (B) of the MAMA-MS-rrl assay developed for the determination of macrolide and lincomycin susceptibility. The heterozygous MYCS-63 (genotype Het; purple line) displayed bimodal melting peak at the specific melting temperatures of genotype L (MYCS-64; dark blue line; Tm 77.27°C) and genotype H (MYCS-65; red line; Tm 82.48°C) *M*. *synoviae* isolates (A). The no-template negative control (light blue line) was not amplified (B), but induced artifact-associated fluorescence (A). Fig 1A.: y-axis: derivative reporter, the negative first-derivative of the normalized fluorescence generated by the reporter during PCR amplification; x-axis: temperature melt curve; Fig 1B.: y-axis: difference between the normalized reporter (Rn) value (normalized fluorescence) of the reaction and the Rn value of the baseline signal generated by the instrument; x-axis: number of cycles.

Besides, the assay MAMA-MS-rrl was able to differentiate heterozygous samples as well: bimodal melting peak at the specific melting temperatures indicated the presence of both nucleotides (A and G) at the position 2054 of the *rrlA*/*B* genes (genotype Het) (Fig 1).

It is important to mention, that isolates which show genotype H with the assay MAMA-MS-parC-2 (i.e. carrying the SNP A253G) cannot be investigated with the assay MAMA-MS-parC-1, as the SNP is at the penultimate position on the binding site of the forward primers parC-1-H and parC-1-L. Thus, as expected, samples MYCS-17, MYCS-20 and MYCS-21 were undetectable with MAMA-MS-parC-1 (S1 Table). Likewise, isolates which showed genotype H with the assay MAMA-MS-rplV-1 (MYCS-79, MYCS-80) were undetectable with the assay MAMA-MS-rplV-2, and vice versa (MYCS-56, MYCS-57, MYCS-71, MYCS-74), as these two assays targeted different nucleotides at the same position (A276C and A276T in the *rplV* gene) (S1 Table).

**Fig 2:**
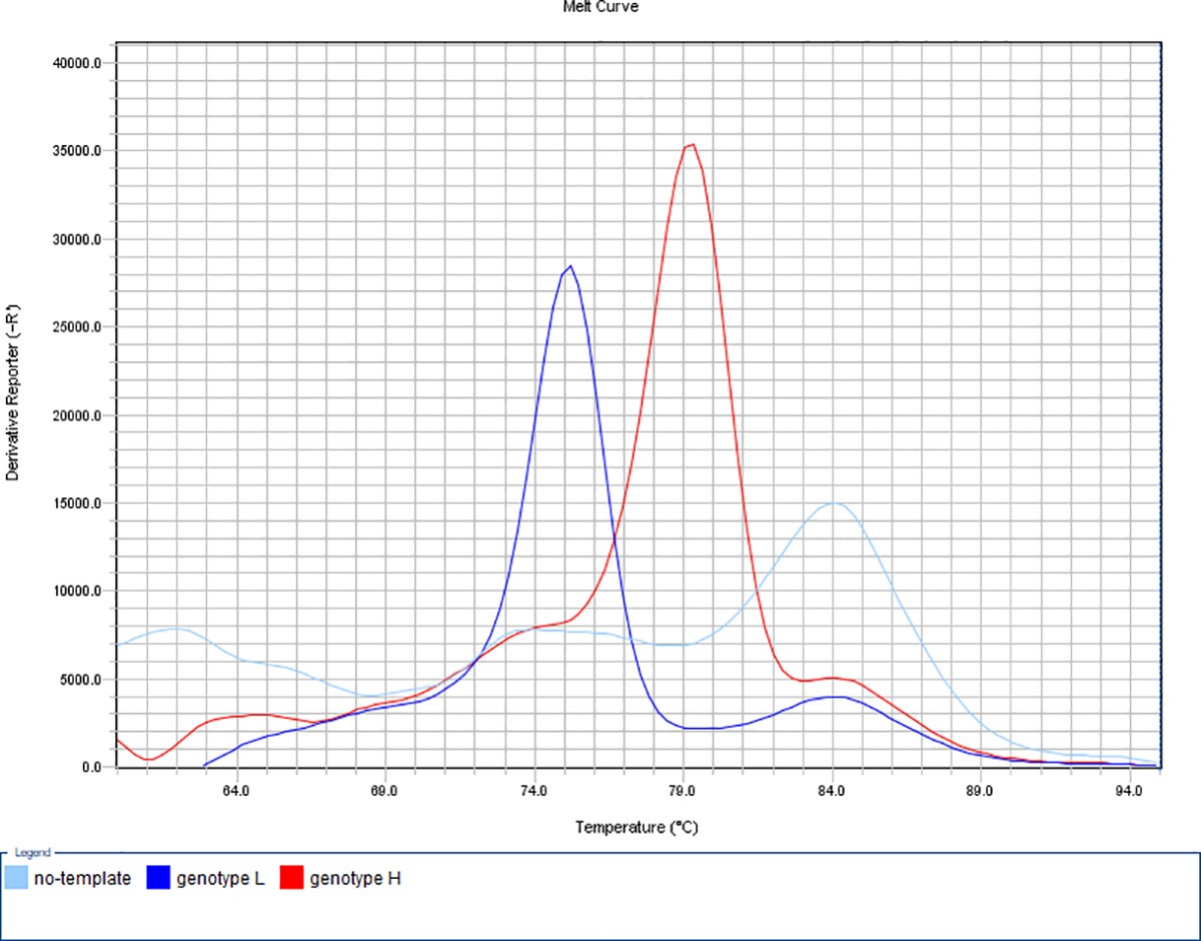
Determination of macrolide susceptibility by the developed MAMA-MS-rplV-1 assay. Amplification products of the genotype L (MYCS-78; dark blue line; Tm 75.48°C) and genotype H (MYCS-79; red line; Tm 79.35°C) *M*. *synoviae* isolates showed specific melting temperatures. The no-template negative control (light blue line) displayed melting peak at non-specific temperature (light blue line; Tm 84.42°C). y-axis: derivative reporter, the negative first-derivative of the normalized fluorescence generated by the reporter during PCR amplification; x-axis: temperature melt curve.

Sizes and melting temperature ranges of the amplicons are listed in Table 2. Slight shifts of the melting temperatures could be detected during the melt curve analysis of PCR amplicons in the independent runs (maximum shift was 2.08°C in MAMA-MS-parC-2), however, the melt curve shapes and temperature differences remained unchanged. Negative controls generated non-specific products with melt-profiles differing from the profiles of the expected two allelic states in the assays MAMA-MS-parC-2 (Tm 71.09°C) and MAMA-MS-rplV-1 (Tm 84.42°C) (Fig 2). In the rest of the assays, negative controls were not amplified (Table 2). Although no-template negative control induced artifact-associated fluorescence in the MAMA-MS-rrl assay (Fig 1A), no quantitation curve can be observed (Fig 1B).

The sensitivity of the melt-MAMA tests changed between 10^2^−10^4^ template copy number/reaction depending on the assay (Table 2). Cross-reactions could be detected only in case of the assay MAMA-MS-rrl with the following avian *Mycoplasma* species: *M*. *anatis*, *M*. *anserisalpingitidis*, *M*. *gallinarum*. Amplicons originating from the DNAs of these species showed melting temperatures around the average Tm of genotype L *M*. *synoviae* strains (77.3°C) (Table 2). In case of clinical specimens containing multiple *Mycoplasma* species [16], only one sample (IDA 52W) had genotype L, which was inconsistent with the MIC data of the *M*. *synoviae* isolate derived from this sample (MYCAV703). Nevertheless, the clinical specimen (IDA 52W) and the *M*. *synoviae* isolate (MYCAV703) showed similar melting temperature (77.27°C and 77.42°C) when investigated with the assay MAMA-MS-rrl.

The five MAMAs developed to determine susceptibility for fluoroquinolones were able to discriminate 79.1% (68/86) of the examined *M*. *synoviae* isolates with elevated MIC values (>1.25 μg/ml) for enrofloxacin or difloxacin. The MAMA-MS-parC-1 targeting the SNP C254T of the *parC* gene was able to detect 63 *M*. *synoviae* isolates with decreased fluoroquinolone susceptibility, while the other targeted mutations of the *gyrA* (A566G), *gyrB* (C1247A), *parC* (A253G) and *parE* (C260T) genes identified 35, 31, 2 and 31 *M*. *synoviae* isolates with elevated fluoroquinolone MIC values, respectively.

In case of lincomycin, molecular detection of the A2054G mutation in the *rrlA/B* genes developed previously for the determination of the nucleotide at this position [9] was able to identify 92.6% (25/27) of the examined *M*. *synoviae* isolates with elevated MIC values (>2 μg/ml). It is worth to mention, that the MIC values (4 μg/ml) against both misclassified samples (MYCS-72 and MYCAV703) were just above the limit.

Except for the same two samples (MYCS-72 and MYCAV703), the three MAMAs developed to determine susceptibility for macrolides were able to discriminate all examined *M*. *synoviae* isolates (94.1%; 32/34) with elevated MIC values for tilmicosin, tylosin or tilvalosin (>8 μg/ml, >1 μg/ml and >0.5 μg/ml, respectively). The MAMA-MS-rrl targeting the SNP A2054G of the *rrlA/B* genes was able to detect 24 *M*. *synoviae* isolates with decreased macrolide susceptibility, while the MAMA-MS-rplV-1 and -2 assays targeting the mutation A276C/T of the *rplV* gene were able to detect 4–4 *M*. *synoviae* isolates with elevated macrolide MIC values.

**Fig 3:**
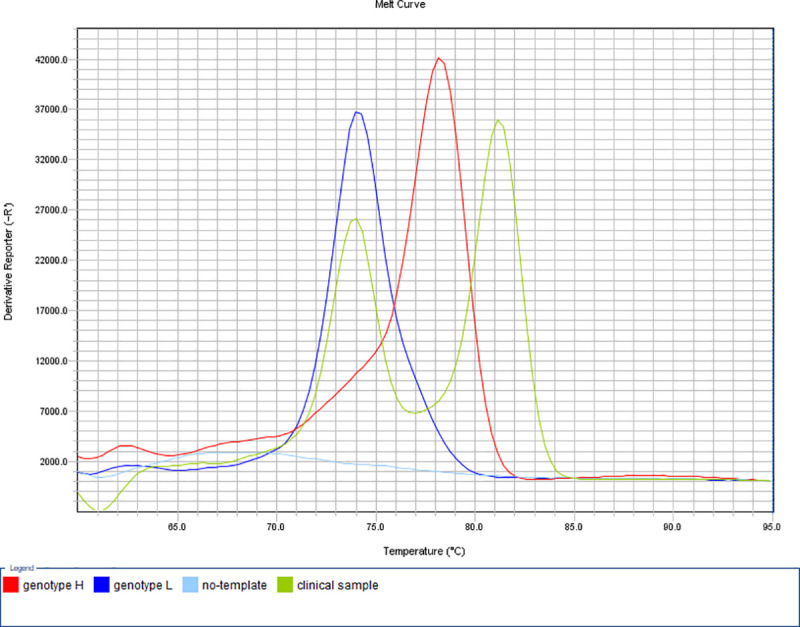
Determination of fluoroquinolone susceptibility by the developed MAMA-MS-gyrA assay. Amplification products of the genotype L (MYCS-23; dark blue line; Tm 74.14°C) and genotype H (MYCS-27; red line; Tm 78.31°C) *M*. *synoviae* isolates showed specific melting temperatures. Clinical sample H1 displayed bimodal curve with a peak at the specific melting temperature of genotype L (Tm 74.02°C) and an additional peak at non-specific temperature (Tm 81.28°C). The no-template negative control (light blue line) was not amplified. y-axis: derivative reporter, the negative first-derivative of the normalized fluorescence generated by the reporter during PCR amplification; x-axis: temperature melt curve.

False result rate of the PCR-based susceptibility tests was 2.9% (3/102) for macrolides and lincomycin, and 14.8% (16/108) for fluoroquinolones, when pure cultures of *M*. *synoviae* isolates were examined. Due to low DNA concentration (CT ≥30.04 by quantitative real-time TaqMan PCR according to Raviv and Kleven [15]), four clinical samples (H1, H2, S4 and P2B) were found to be under the detection limit of certain assays. Apart from this, clinical samples showed identical genotype calls with the *M*. *synoviae* isolates derived from the corresponding specimens by all of the designed MAMA tests. However, non-specific melting curves could be observed beside the genotype-specific melting temperatures, resulting in bimodal melting peaks in a few cases of clinical samples containing the DNA of *M*. *synoviae* only or multiple *Mycoplasma* species as well [16] (Fig 3).

As no discrepancy could be observed between the genotype calls of *M*. *synoviae* isolates and their corresponding clinical specimens, false result rate of the PCR-based susceptibility tests has not been calculated specifically for clinical samples.

## Discussion

Determination of antibiotic susceptibility in mycoplasmas by the conventional *in vitro* techniques is very labor-intensive and time-consuming method, and the interpretation of the results is difficult as well. Thus, there is an increasing need for reliable, rapid and convenient antimicrobial susceptibility tests in order to support the choice of the most appropriate antibiotic therapy. In the current study, molecular biological assays were designed for the simultaneous detection of markers responsible for high MICs to certain antibiotics in *M*. *synoviae*.

Assays developed in the present study reliably discriminated *M*. *synoviae* genotypes characterized by low or high MICs to the tested antibiotics by the melting temperatures of their amplicons. However, the possible presence of unexplored mutations within the binding sites of the primers or unknown variable mutation events may influence the reliability of the methods, and other resistance mechanisms may also complicate the determination of antibiotic susceptibility [20, 21].

Although most *M*. *synoviae* isolates with decreased fluoroquinolone susceptibility was detected by the assay MAMA-MS-parC-1 targeting the SNP C254T of the *parC* gene, it is advisable to investigate the presence of the other potentially resistance-related mutations of the *gyrA* (A566G), *gyrB* (C1247A), *parC* (A253G) and *parE* (C260T) genes by the designed MAMAs as well to further enhance the reliability of the results by simultaneous detection of several mutations in different genes. Besides, performing all of these assays can provide information about the prevalence of these resistance-related mutations, which can be later used for reducing the number of the tests, or setting up an order according to their relevance. However, as isolates which show genotype H with the assay MAMA-MS-parC-2 are found to be uncommon, and they are undetectable with the assay MAMA-MS-parC-1, it is advisable to perform the assay MAMA-MS-parC-2 just in the case when a sample cannot be amplified during the MAMA-MS-parC-1 assay.

Most *M*. *synoviae* isolates with decreased macrolide and lincomycin susceptibility was detected by the assay MAMA-MS-rrl targeting the SNP A2054G of the *rrlA/B* genes. Nonetheless, performing the assays MAMA-MS-rplV-1 and -2 targeting the mutation A276C/T of the *rplV* gene beside the MAMA-MS-rrl is highly recommended for the determination of macrolide susceptibility, as the mutations A276C/T occurred exclusively in isolates lacking the SNP A2054G of the *rrl* genes. However, as isolates which show genotype H with the assay MAMA-MS-rplV-1 are undetectable with the assay MAMA-MS-rplV-2, and vice versa, it is enough to apply one of these two assays at the first step, and perform the other one just in the case when a sample cannot be amplified. Mutation A276T was found to be more frequent according to the previous results of investigating whole genome sequenced *M*. *synoviae* strains [9], while only the presence of the A276C was observed in the clinical sample-derived isolates examined in this study. Thus, priorities of these two assays are hard to set for the time being.

The 20 examined clinical samples showed identical genotype calls with the 20 *M*. *synoviae* isolates derived from the corresponding specimens by each assay which had sufficient sensitivity for the detection, including samples (n = 10) originating from birds with mixed infections of different *Mycoplasma* species [16] as well. Thus, the assays could be considered applicable on DNA samples extracted directly from clinical specimens. However, a limitation of this method is that some of the developed assays do not have the sensitivity to test samples with low specific DNA load (Ct >30 according to Raviv and Kleven [15]). Therefore, these molecular methods are most likely to be successful, when investigating clinical specimens collected during the acute phase of the infection. In the later stages, the DNA concentration of the samples can decrease due to the reduced number of the bacteria. In these cases, isolation is recommended instead of testing DNAs extracted directly from clinical samples. Cross-reactions with other *Mycoplasma* species may occur as well in the case of assay MAMA-MS-rrl when testing clinical specimens. As the amplicons of these species had melting temperatures around the average Tm of genotype L *M*. *synoviae* strains (77.3°C), excluding the presence of cross-reacting *Mycoplasma* species by a universal *Mycoplasma* PCR in the doubtful cases is recommended for the reliable determination of macrolide and lincomycin susceptibility of *M*. *synoviae* isolates.

The powerful advantages of the described method are rapidity, convenience and cost-effectiveness. In the current study, all PCRs were designed with the same thermal profile allowing their simultaneous application, and the developed MAMAs can be performed even on basic real-time PCR machines. Thus, the application of our approach provides a feasible tool for diagnostics.

## Conclusion

Supporting the results of conventional *in vitro* sensitivity tests, the molecular biological assays developed in this study can provide excellent assistance for the choice of the most appropriate antibiotic therapy. The use of this molecular tool can contribute to achieve therapeutic success by reducing the detection time of antibiotic susceptibility, thereby significantly reduce economic losses. Furthermore, early prediction of antibiotic efficacy by these assays can support prudent antibiotic usage instead of empirical treatment, and decrease public health concerns related to bacterial resistance by playing a proactive role in the reduction of antibiotics use.

## Supporting information

S1 Table Background information and MIC data of the used samples, and results of the developed MAMA tests. A and B: Background information of the 92 *M*. *synoviae* strains provided by previous studies [9; 12] and results of fluoroquinolone (A), macrolide and lincomycin (B) susceptibility determination by broth microdilution and the developed melt-MAMA tests. C and D: Background information of the 20 clinical specimens and 20 clinical sample-derived *M*. *synoviae* isolates examined in this study and results of fluoroquinolone (C), macrolide and lincomycin (D) susceptibility determination by broth microdilution and the developed melt-MAMA tests. MIC values were considered to be elevated above 1.25 μg/ml for enrofloxacin and difloxacin; 8 μg/ml for tilmicosin; 1 μg/ml for tylosin; 0.5 μg/ml for tylvalosin; and 2 μg/ml for lincomycin. Color codes indicating: elevated MIC (dark grey); low MIC (light grey); genotype assignment is congruent with the MIC data (green); genotype assignment is incongruent with the MIC data, i.e. false result (yellow); congruency cannot be evaluated due to that the sample was below detection limit in some of the assays (blue); excluded from the evaluation due to missing MIC data (no color). PCR: polymerase chain reaction; CT: cycle threshold; MAMA: mismatch amplification mutation assay; MIC: minimal inhibitory concentration; Gt: genotype according to whole genome sequence; Tm: melting temperature; n.t.: not tested; n.a.: not available; L: genotype characterized by low MIC values; H: genotype characterized by high MIC values; Het: heterozygous genotype; NS: non-specific melting temperature; -: undetected; *: expected to be undetectable by the assay
(XLSX)
